# Prediction of response to repetitive transcranial magnetic stimulation in phantom sounds based on individual brain anatomy

**DOI:** 10.1093/braincomms/fcab115

**Published:** 2021-05-27

**Authors:** Timm B Poeppl, Martin Schecklmann, Katrin Sakreida, Michael Landgrebe, Berthold Langguth, Simon B Eickhoff

**Affiliations:** 1 Department of Psychiatry, Psychotherapy and Psychosomatics, Faculty of Medicine, RWTH Aachen University, Aachen, Germany; 2 Department of Psychiatry and Psychotherapy, Universität Regensburg, Regensburg, Germany; 3 Department of Psychiatry, Psychotherapy and Psychosomatics, kbo-Lech-Mangfall-Klinik Agatharied, Hausham, Germany; 4 Institute for Systems Neuroscience, Heinrich Heine University, Düsseldorf, Germany; 5 Institute of Neuroscience and Medicine, Brain and Behaviour (INM-7), Research Centre Jülich, Jülich, Germany

**Keywords:** phantom sounds, prediction, machine learning, magnetic resonance imaging, tinnitus

## Abstract

Non-invasive brain stimulation can reduce the severity of tinnitus phantom sounds beyond the time of stimulation by inducing regional neuroplastic changes. However, there are no good clinical predictors for treatment outcome. We used machine learning to investigate whether brain anatomy can predict therapeutic outcome. Sixty-one chronic tinnitus patients received repetitive transcranial magnetic stimulation of left dorsolateral prefrontal and temporal cortex. Before repetitive transcranial magnetic stimulation, a structural magnetic resonance image was obtained from all patients. To predict individual treatment response in new subjects, we employed a support vector machine ensemble for individual out-of-sample prediction. In the cross-validation, the support vector machine ensemble based on stratified sub-sampling and feature selection yielded an area under the curve of 0.87 for prediction of therapy success in new, previously unseen subjects. This corresponded to a balanced accuracy of 83.5%, sensitivity of 77.2% and specificity of 87.2%. Investigating the most selected features showed the involvement of the auditory cortex but also revealed a network of non-auditory brain areas. These findings suggest that idiosyncratic brain patterns accurately predict individual responses to repetitive transcranial magnetic stimulation treatment for tinnitus. Our findings may hence pave the way for future investigations into the precision treatment of tinnitus, involving automatic identification of the appropriate treatment method for the individual patient.

## Teaser

Poeppl et al. applied machine-learning algorithms to structural MRI for prediction of response to non-invasive brain stimulation in patients with tinnitus phantom sounds. The identified classifier predicted therapy success with an accuracy of 83.5%. The findings may hence pave the way for future investigations into precision treatment of tinnitus.

## Graphical Abstract

**Graphical Abstract:**
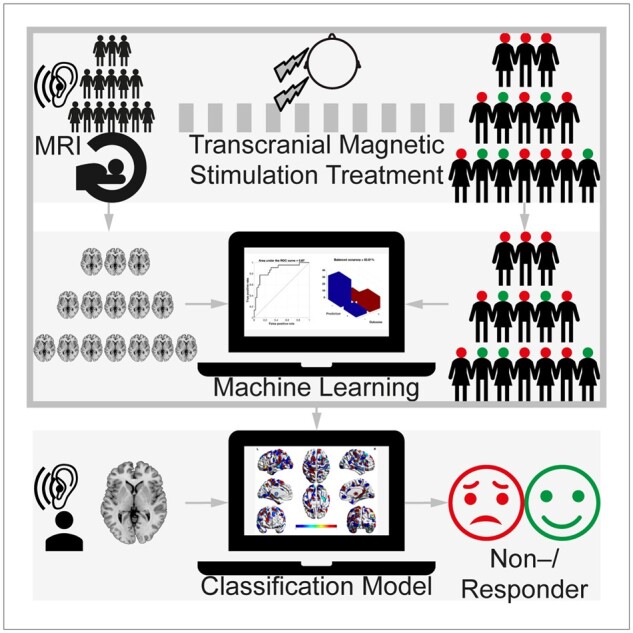

## Introduction

Tinnitus, the perception of sound in absence of a corresponding external acoustic stimulus, is an excellent paradigm to gain insight into neural mechanisms of phantom sensations.^1^^,^^2^ Unlike auditory hallucinations, tinnitus is an auditory phantom phenomenon of unformed acoustic nature, usually described as ringing, hissing or buzzing.^3^ Neural alterations underlying these non-psychotic phantom perceptions involve structure, function and connectivity of not only auditory but also non-auditory brain regions.^2^^,^^4^ Particularly a dysregulation of limbic and auditory networks might contribute to tinnitus pathophysiology.^5^

Given its estimated prevalence of 10–15% in the general population, tinnitus is considered a common disorder, entailing severe impairments in the quality of life for around 10–20% of the affected.^3^ As no effective and specific pharmacological treatment is available, therapeutic options are limited.^6^ Besides hearing aids in cases of concomitant hearing loss, the most effective approaches to ameliorate tinnitus symptoms are sound therapy and cognitive behavioural therapy.^6^ Recently, the use of non-invasive brain stimulation techniques has gained considerable momentum in the treatment of tinnitus. A meta-analysis on the effect of rTMS in randomized, placebo-controlled trials indicated medium to large effect sizes for reducing tinnitus.^7^ These results support the potential of rTMS to modulate auditory phantom phenomena, pointing to a new option for a yet hardly treatable disorder. Yet, the observation that some subjects do not respond to this treatment at all remains a major challenge to further clinical investigation and ultimately application.^7^

Complicating matters, no useful demographic or clinical predictors of rTMS treatment outcome have been identified so far.^8^ Success may thus depend on the specific patient’s neurobiological properties.^9^ Using longitudinal sMRI, we indeed found evidence that treatment response to non-invasive brain stimulation in tinnitus is linked to neuroplastic changes that affect brain structure and connectivity of the lateral prefrontal, operculo-insular as well as inferior temporal cortex.^9^ These results provided insight into the rTMS-induced plastic processes underlying therapeutic response on a group level. However, the findings did not allow for an *individual* prediction of therapeutic outcomes. That is, while previous findings may provide insight into mechanisms underlying therapeutic response, they unfortunately do not represent a predictive biomarker.

Here, we used multivariate pattern analysis to test whether brain anatomy as assessed by sMRI allows to predict therapeutic outcome in individual patients that were not part of the training set. To this end, we obtained standardized images from tinnitus patients before application of rTMS based on a protocol yielding significantly more treatment responders than sham-stimulation and previous stimulation protocols.^10^^,^^11^ Employing an SVM ensemble, we assessed whether treatment response can be predicted by brain morphology before the intervention.

## Materials and methods

### Subjects

We recruited a cohort of rTMS-naïve subjects with chronic subjective tinnitus. All patients provided written informed consent to participate in the study. The study protocol was approved by the local ethics committee. MRI was performed immediately before the first of all 10 rTMS sessions that subjects underwent on 10 consecutive working days. No subject received concomitant psychotropic medication. Treatment response was defined according to current standards on the basis of previously calculated minimal clinically important difference.^12^ More specifically, classification as ‘responder’ presupposed a reduction by at least 5 points on the Tinnitus Questionnaire, a validated and commonly used instrument for the assessment of tinnitus severity.^12^^,^^13^ Based on this criterion, 22 (36%) patients were classified as responders and 39 (64%) patients as non-responders. There were no baseline differences in age, sex, hearing loss, tinnitus laterality, tinnitus duration or tinnitus severity between both groups (Table 1).

**Table 1:** Patients’ characteristics

	Non-responders	Responders	*P*-value
Subjects (*N*)	39	22	N/A
Age (years)	52.5 ± 9.2	52.1 ± 12.4	0.881
Sex (male/female)	33/6	15/7	0.132
Hearing loss (dB)^a^	21.0 ± 13.2	19.6 ± 12.1	0.743
Tinnitus laterality (L/R/B)^b^	13/15/11	5/13/3	0.207
Tinnitus duration (months)^c^	76.2 ± 79.2	98.4 ± 117.0	0.398
Tinnitus severity (TQ)	47.7 ± 19.8	44.4 ± 16.0	0.503
Tinnitus change after rTMS (TQ)	2.0 ± 4.5	−10.2 ± 4.9	<0.001

Values are reported as mean ± standard deviation. *P-*values were determined by a two-sample *t-*test for age, hearing loss, tinnitus duration and tinnitus severity and a χ^2^ test of independence for sex and tinnitus laterality.^13^

a Data available for 28/13 non-/responders.

b Data available for 39/21 non-/responders.

c Data available for 37/20 non-/responders.

### rTMS

After determination of the resting motor threshold (Supplementary material), in each of the subsequent 10 sessions, patients received rTMS of the left dorsolateral prefrontal cortex (40 trains with 50 stimuli; 25 s intertrain interval; 20 Hz; 110% resting motor threshold), immediately followed by low-frequency rTMS (2000 stimuli; 1 Hz; 110% resting motor threshold) of the left temporal cortex. In addition to the auditory network, this protocol thus targets the dorsolateral prefrontal cortex, given electrophysiological evidence that tinnitus might result from dysfunctional top-down inhibiton.^14^

### sMRI and data pre-processing

After visual inspection for quality assurance, T1-weighted MRI scans were processed using the Computational Anatomy Toolbox (Supplementary material). One challenge when using machine-learning approaches on brain imaging data such as the voxel-based morphometry information available here, is the high (nominal) dimensionality of the data consisting of more than 200 000 individual voxels. This creates a very poor feature-to-sample ratio, which is detrimental to any effective model training in terms of computing time and performance. To overcome this challenge, we followed an atlas-based strategy for data representation, capitalizing on the fact that the brain is topographically organized into distinct areas of largely homogeneous structural and functional properties.^15^ This approach provides a biologically informed compression and hence feature reduction. Here, we used 673 grey matter parcels from well-validated brain parcellations (600 cortical parcels from Schaefer et al.,^16^ 36 subcortical grey matter parcels from Fan et al.,^17^ and 37 cerebellar parcels from Buckner et al.^18^). Thus, a subject’s individual grey matter anatomy was represented by 673 features, each reflecting the winsorized mean of the voxel-wise GMV values for each area.

### Statistical analyses

To investigate mass-univariate differences in brain structure between responders and non-responders, (parcel-wise) GMVs were compared by means of an ANOVA accounting for age and sex. Inference was performed by non-parametric, permutation-based thresholding at a FWE corrected *P* < 0.05.

In order to predict treatment effects, we employed an SVM ensemble. Model performance was assessed using leave-one-out cross-validation, i.e., based on the classification accuracy in patients that have not been seen by the algorithm during model training. More specifically, among the training set, we randomly sampled the same number of responding and non-responding patients. Sampling was performed without replacement at 95% of the smaller group within the training set. That is, if the training set contained 39 patients that did not respond and 21 that did, we randomly sampled 20 patients from either group. Among the sampled subjects, we then performed feature selection, only retaining the top 5% based on the univariate difference in the selected subset of the training data. Based on the hereby selected features, we then fitted an SVM model using the LIBSVM library^19^ (radial basis function, C = 1) on the randomly sampled subset of the training data. This model was then evaluated on the held-out test data. Repeating this procedure 50 000 times and computing the median of the resulting decision values (indicating distance and direction from the hyperplane) then resulted in the final prediction.

### Data availability statement

The data that support the findings of this study are available from the corresponding author, upon reasonable request.

## Results

In the mass-univariate approach, we found no significant differences in GMV between responders and non-responders (*P* < 0.05, FWE). At an uncorrected threshold of *P* < 0.05, widespread differences were observed. Responders exhibited more GMV in the left and right superior temporal cortex, left fusiform cortex, left occipital cortex, the left superior parietal and bilateral inferior parietal lobules, left insula, right operculum, bilateral premotor cortex, left ventrolateral and right dorsomedial prefrontal cortex. In contrast, non-responders had more GMV in the left dorsolateral prefrontal cortex, left cerebellum, left (para-)hippocampus and left superior parietal lobule (Supplementary Fig. 1).

**Figure 1:**
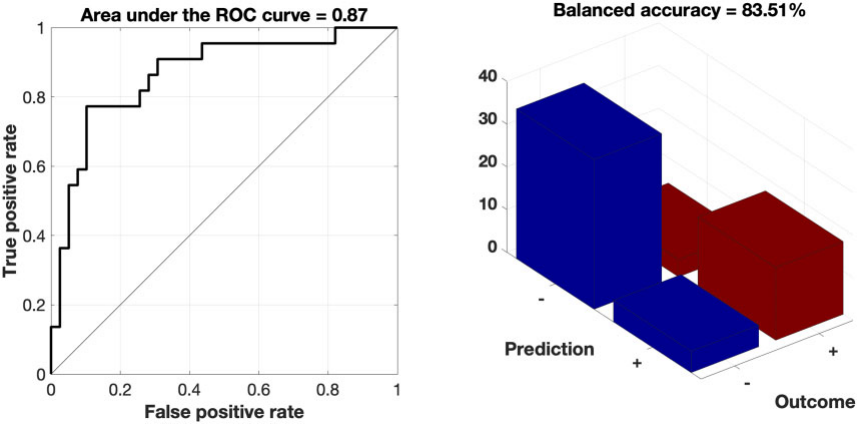
Performance of the classifier. Our SVM ensemble yielded an area under the curve of 0.87 for the prediction of individual therapeutic success (left). That is, on the basis of a whole-brain parcellation of grey matter, the machine learning algorithm predicted individual response to rTMS in tinnitus patients with an accuracy of 83.5% (right).

We fitted a supervised pattern extraction algorithm based on variation in GMV from 673 target regions using SVM models. In the cross-validation, the SVM ensemble based on stratified subsampling yielded an area under the curve of 0.87 for the prediction of therapy success in new, previously unseen subjects (Fig. 1). That is, our machine learning-based outcome classifier trained on standard sMRI data correctly separated future responders from non-responders before treatment with a cross-validated balanced accuracy of 83.5%. This corresponded to a sensitivity of 77.2% and a specificity of 87.2% (Fig. 1). Hence, the positive likelihood ratio was 6.0. A positive/negative prediction thus increased a patient’s response/non-response likelihood by +47.4%/+19.6%, adding up to a total gain in prognostic accuracy of +67.0%. Finally, the F1-score (harmonic mean of sensitivity and positive predictive value) was 0.77. Of note, sociodemographic, clinical, or psychopharmacological characteristics did not bias the rTMS outcome classifier’s stratification effects (Table 1).

**Figure 2:**
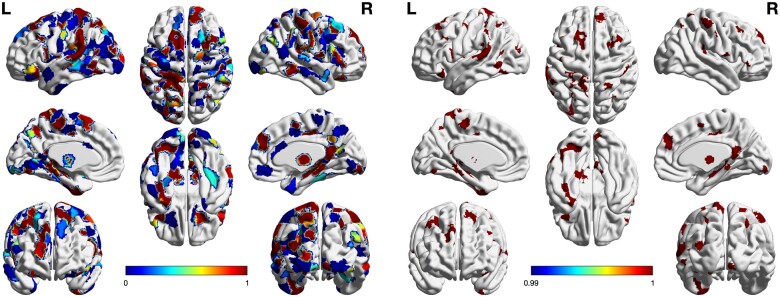
Overview of selected features. The machine learning model was fitted on the depicted grey matter features from randomly subsets of the training data. The selection included auditory but also various non-auditory brain areas (left panel). Restriction to the most frequently (i.e. in at least 99% of the samples) selected regions revealed that the selection pattern was highly consistent. This pattern consisted of anatomical features of lateral and medial prefrontal cortex, opercular cortex and postcentral gyrus, occipitotemporal cortex, superior temporal cortex, pallidum, thalamus and cerebellum (right panel). Colours indicate the frequency of being selected.

Investigating the most frequently selected regions revealed auditory areas such as Heschl’s gyrus and planum temporale but also many areas throughout the brain (Fig. 2). Focusing on the regions that were selected in at least 99% of the samples indicated high consistency in the selection pattern. This pattern of the most predictive regions was hence robust, yet complex and included a variety of regions in the prefrontal, parietal, temporal and occipital cortex as well as in the diencephalon and cerebellum (Fig. 2).

Investigating how the selected regions differed between groups, we found that the neuroanatomical pattern predicting subsequent response to rTMS particularly involved regions with relative grey matter increase in the parietal cortex, premotor cortex, superior temporal cortex as well as in the lateral and dorsomedial prefrontal cortex. Four left-hemispheric regions of the predictive pattern in the cerebellum, superior parietal lobule, (para)hippocampus and dorsolateral prefrontal cortex were characterized by a grey matter decrease.

In addition, we fitted a supervised pattern extraction algorithm based on sociodemographic and clinical characteristics (i.e. age, sex, hearing loss as well as tinnitus laterality, duration and severity; cf., Table 1) using analogous SVM models. In the cross-validation, this SVM ensemble based on stratified subsampling yielded an area under the curve of 0.55 for the prediction of therapy success in new, previously unseen subjects (Supplementary Fig. 2). That is, our machine learning-based outcome classifier trained on non-imaging data correctly separated future responders from non-responders before treatment with a cross-validated balanced accuracy of 62.2%. A positive/negative prediction thus increased a patient’s response/non-response likelihood by +25.1%/−2.7%, adding up to a total gain in prognostic accuracy of +22.4%.

## Discussion

This is the first study reporting the successful application of MRI-based machine learning for the prediction of individual future responses to rTMS treatment of phantom sounds. Our imaging-based model accurately predicted therapeutic outcomes in 8–9 of 10 patients. In contrast, the non-imaging-based model was about 20% less accurate. That is, clinical information including tinnitus characteristics is helpful but outperformed by neurobiological information in response prediction. This finding may indicate that therapeutic response mainly depends on neuroplastic capabilities of brain regions forming a complex pattern. The relative grey matter increases in parietal, premotor, auditory, ventrolateral and dorsomedial prefrontal cortices as well as the reductions in cerebellum, superior parietal lobule, (para)hippocampus and dorsolateral prefrontal cortex before treatment may mediate subsequent tinnitus alleviation induced by rTMS to the left dorsolateral prefrontal and temporal cortex. This neuroanatomical baseline variance in tinnitus patients hence seems to represent a critical biological factor determining the efficacy of rTMS in improving phantom sounds.

Intriguingly, the mass-univariate approach was not able to discriminate responders from non-responders before rTMS treatment with statistical significance but our multivariate pattern learning algorithm could detect a brain configuration predicting individual response to rTMS with high accuracy. This is in line with the observation that brain patterns predicting response to rTMS are highly complex and vary between other diseases such as depression and schizophrenia.^20^^,^^21^ The complexity of the identified classifier may also (partly) result from the known high variability of the neural substrate underlying tinnitus,^4^ which in turn seems to be reflected in both neurobiological and clinical subtypes.^22^^,^^23^ Moreover, this complex, predictive formation involving auditory and non-auditory regions reinforces the implication of the latter in phantom sounds and may explain the lack of clinical predictors for rTMS treatment outcome. Taken together, the identified predictive brain pattern most likely represents an interaction of general responsivity to rTMS with tinnitus phenotypes.

Notably, this anatomical grey matter pattern overlaps with regions that show rTMS-induced neuroplastic changes underlying therapeutic response in tinnitus patients.^9^ These include left dorsolateral prefrontal, left operculo-insular and right inferior temporal cortex. However, our machine-learning classifier consistently selected also a variety of other (non-)auditory regions to predict therapeutic response with high accuracy. Hence, not only regions showing longitudinal response-related structural changes but rather their complex composite configuration with several other brain regions determines the therapeutic outcome and can thus serve as a biomarker. This observation matches with analogous results from other diseases such as schizophrenia, where a complex pattern of baseline grey matter variation predicts rTMS-induced improvement of negative symptoms, but the plastic structural response of only a few regions is linked to symptom improvement.^21^^,^^24^ Similarly, response to electroconvulsive therapy in depression can be predicted by a relatively complex structural grey matter pattern but is merely associated with longitudinal changes predominantly in the hippocampal formation.^25^ That is, our findings match with the notion that across different diseases and different non-invasive brain stimulation approaches, structural baseline brain patterns predicting response are intricate, while response-associated longitudinal changes are rather straightforward.

Still, the detailed link between the classifier’s mesoscopic structural brain pattern and the putative underlying mechanistic surrogate remains incomplete. Parallel investigations using diacritic MRI protocols may specify contributions of histopathological properties and thus reveal the microscopic mechanisms underlying the observed mesoscopic brain pattern, which determines the individual capability to respond to rTMS. However, positron emission tomography studies showed that high-frequency rTMS of the left dorsolateral prefrontal cortex modulates dopamine release in other prefrontal regions.^26^ Moreover, there is evidence that low-frequency rTMS affects GABAergic neurotransmission not only in the stimulated brain region but also in various regions widely spread over the cortex.^27^ Given the association of tinnitus with a reduced γ-aminobutyric acid (GABA) concentration in theauditory cortex and changes in tinnitus perception by pharmacologically modulating the auditolimbic dopaminergic pathway,^28^^,^^29^ corresponding rTMS protocols as implemented in our study might reduce tinnitus by manipulating neurochemistry in candidate regions. It seems thus plausible that the baseline configuration of the respective regions determines the(ir) responsivity to rTMS. However, it also has to be noted that prediction does not need causal relationships.

Given response rates between 30–40% and costs of ≈2000 USD per patient for a course of rTMS,^10^^,^^30^ usually paid out of the patient’s pocket, a reliable predictor of therapeutic response is also economically relevant. In addition, its availability could save time by providing patients with another treatment already at an early stage and obviate raising false hopes and futile expenditures. Certainly, our biomarker is based on MRI, which is not free of charge. However, an MRI of the brain is part of clinical standard diagnostics in tinnitus and does therefore not imply additional expenses. In total, the use of our machine learning-derived predictor increases prognostic accuracy by +67.0%. For direct clinical translation and broad application, further prospective studies should investigate its stability over prevalent MRI machines and various T1-weighted sequences that reflect the clinical reality when a tinnitus patient is referred to rTMS treatment and brings his brain MRI from a radiological practice.

In conclusion, we showed that individual responses to rTMS treatment for tinnitus may be accurately predicted using biomarkers based on structural neuroimaging. The results provide a robust basis for the development of personalized rTMS interventions taking into account individual neurobiology. Our findings may hence pave the way for future investigations into precision tinnitus treatment involving automatic identification of the appropriate treatment method for the individual patient suffering from this multifaceted phenomenon.

## Supplementary material

Supplementary material is available at *Brain Communications* online.

## Funding

S.B.E. was supported by the Deutsche Forschungsgemeinschaft (DFG, EI 816/21–1), the National Institute of Mental Health (R01-MH074457), the Helmholtz Portfolio Theme ‘Supercomputing and Modeling for the Human Brain’ and the European Union’s Horizon 2020 Research and Innovation Programme under Grant Agreement No. 945539 (HBP SGA3).

## Competing interests

The authors report no competing interests.

## Supplementary Material

fcab115_Supplementary_Data

## Abbreviations

B =: bilateral
FWE =: family-wise error
GMV =: grey matter volume
L =: left
N/A =: not applicable
R =: right
ROC =: receiver operating characteristic
rTMS =: repetitive transcranial magnetic stimulation
sMRI =: structural magnetic resonance imaging
SVM =: support vector machine
TQ =: Tinnitus Questionnaire
