# Supplementary material for: Prediction of response to repetitive transcranial magnetic stimulation in phantom sounds based on individual brain anatomy
Source: Brain Commun. 2021 May 27;3(3):fcab115. doi: 10.1093/braincomms/fcab115 (PMC8361389; doi:10.1093/braincomms/fcab115)
Supplement: fcab115_Supplementary_Data [file fcab115_supplementary_data.docx]

**Supplementary material**

**Supplementary methods**

**Repetitive transcranial magnetic stimulation**

Initially, the resting motor threshold (RMT) was determined for the right M. abductor digiti minimi and defined as the lowest intensity at which at least four of eight consecutive magnetically evoked potentials were ≥ 50 mV in amplitude while the investigated muscle was at rest. Coil positioning over the temporal cortex was based on 10–20 EEG coordinates and over the left dorsolateral prefrontal cortex on a standard algorithm by moving the coil 6 cm from the RMT hot spot of the right M. abductor digiti minimi in anterior direction.^10^

**Structural magnetic resonance imaging and data preprocessing**

Structural MRI scans were acquired on a 1.5 T scanner (MAGNETOM Sonata, Siemens Medical Solutions, Erlangen, Germany) equipped with a standard 8-channel birdcage head coil. T1-weighted images were obtained using a 3D magnetization-prepared rapid acquisition with gradient echo sequence (repetition time 1880 ms, echo time 3.42 ms, flip angle 15°, matrix size 256 × 256, 176 sagittal slices, voxel size 1 × 1 × 1 mm^3^).

Preprocessing with the toolbox (CAT12, http://www.neuro.uni-jena.de/cat/) included bias-field correction, noise removal and skull stripping, normalization to Montreal Neurological Institute (MNI) space and tissue segmentation. The ensuing grey matter density maps in standard space are then modulated, i.e., scaled by the amount of volume changes needed to match the individual brain to the template by non-linear registration. In the modulated images, each voxel thus represents the local grey matter volume (GMV) of the corresponding location in the individual brain.

**Supplementary figures**

**Supplementary figure 1**

**
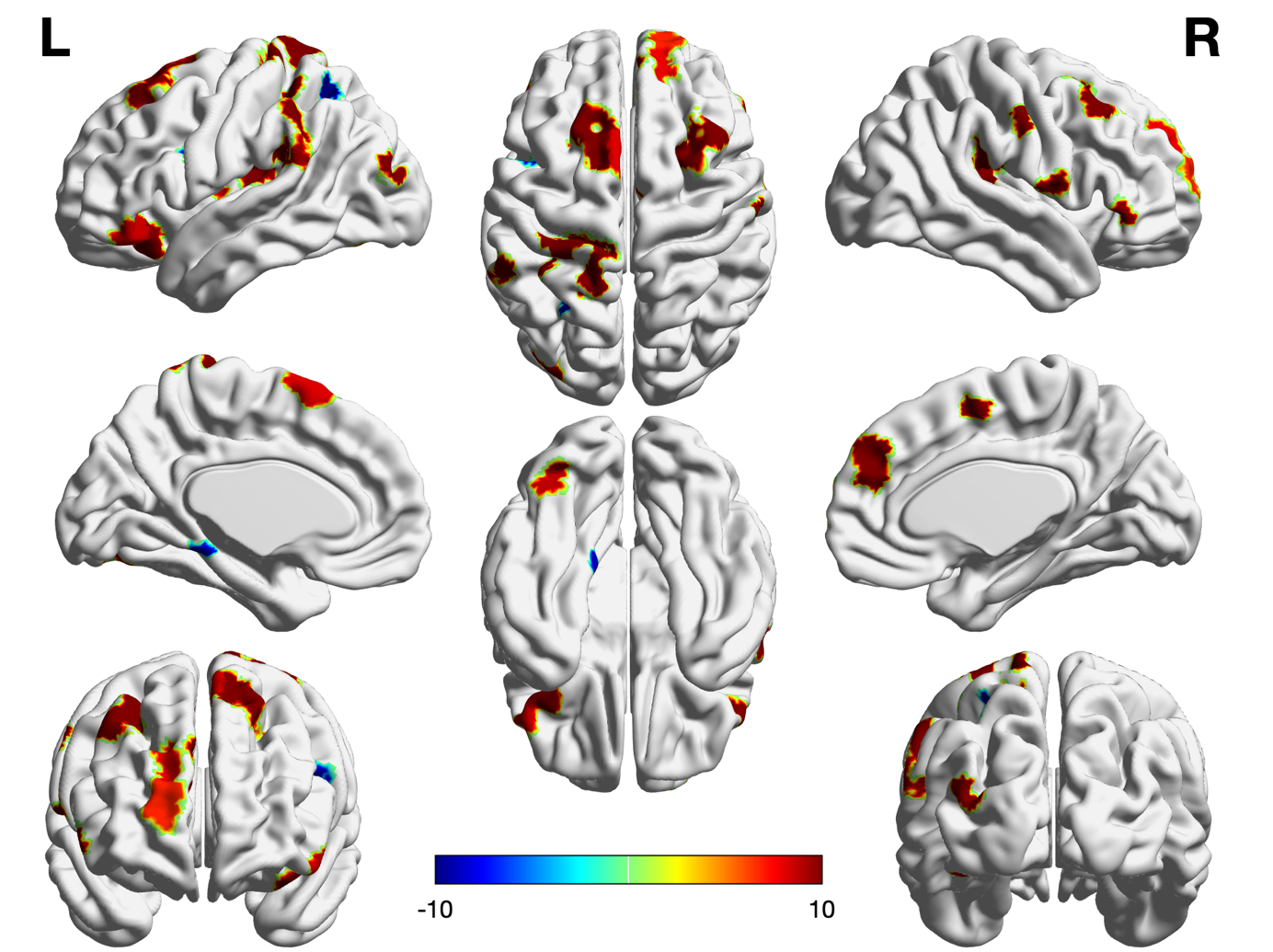
**

**Mass-univariate comparison of gray matter morphology.** Responders (red) exhibited more gray matter volume (GMV) in the superior temporal cortex, fusiform cortex, occipital cortex, superior parietal and bilateral inferior parietal lobules, insula, premotor cortex as well as left ventrolateral and right dorsomedial prefrontal cortex. In contrast, nonresponders (blue) had more GMV in the cerebellum, superior parietal lobule, (para-)hippocampus, and dorsolateral prefrontal cortex (p<0.05, uncorrected).

Gradients of the color bars represent percent change in GMV. L, left; R, right.

**Supplementary figure 2**


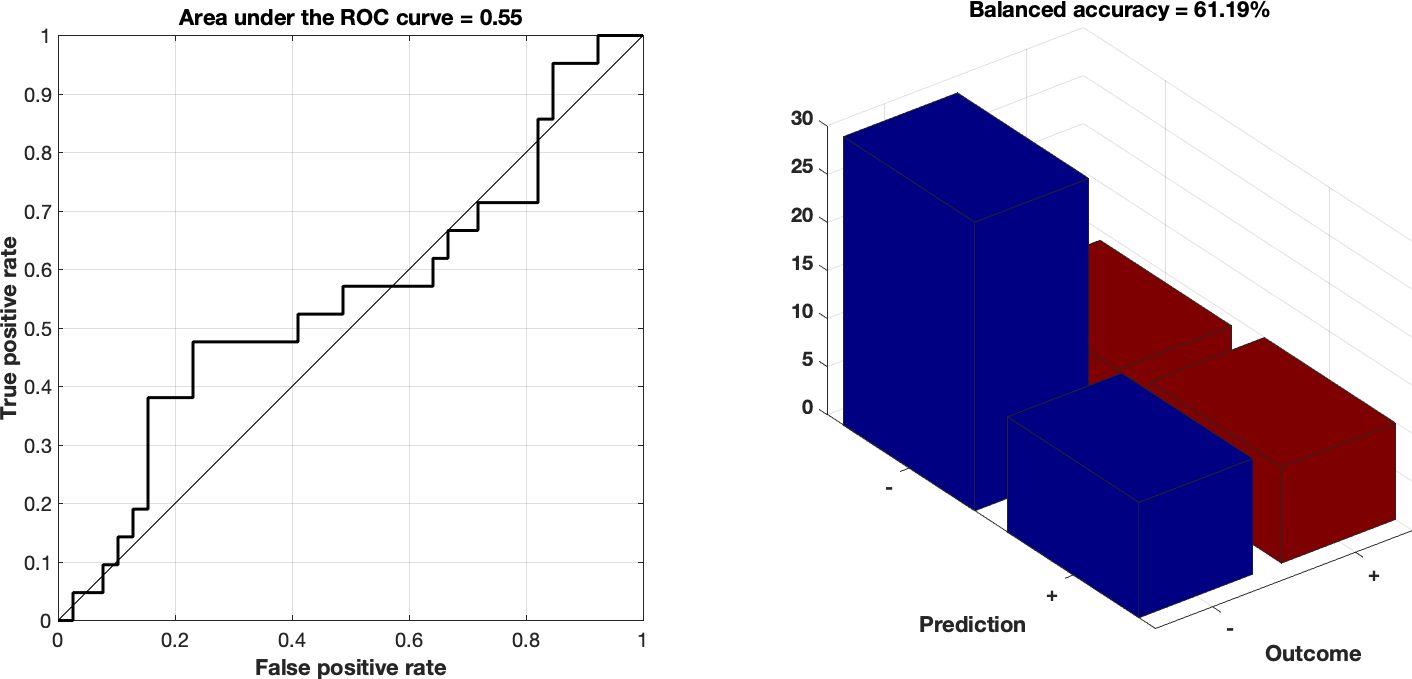


**Performance of the nonimaging classifier.** Our support vector machine ensemble yielded an area under the curve of 0.55 for the prediction of individual therapeutic success (left). That is, on the basis of sociodemographic and clinical characteristics, the machine learning algorithm predicted individual response to repetitive transcranial magnetic stimulation in tinnitus patients with an accuracy of 61.2% (right).
